# Detection of HIV transmission clusters from phylogenetic trees using a multi-state birth–death model

**DOI:** 10.1098/rsif.2018.0512

**Published:** 2018-09-05

**Authors:** Joëlle Barido-Sottani, Timothy G. Vaughan, Tanja Stadler

**Affiliations:** 1 Department of Biosystems Science and Engineering, ETH Zürich, Basel, Switzerland; 2 Swiss Institute of Bioinformatics (SIB), Switzerland

**Keywords:** phylodynamics, multi-state birth–death model, transmission clusters, maximum likelihood

## Abstract

HIV patients form clusters in HIV transmission networks. Accurate identification of these transmission clusters is essential to effectively target public health interventions. One reason for clustering is that the underlying contact network contains many local communities. We present a new maximum-likelihood method for identifying transmission clusters caused by community structure, based on phylogenetic trees. The method employs a multi-state birth–death (MSBD) model which detects changes in transmission rate, which are interpreted as the introduction of the epidemic into a new susceptible community, i.e. the formation of a new cluster. We show that the MSBD method is able to reliably infer the clusters and the transmission parameters from a pathogen phylogeny based on our simulations. In contrast to existing cutpoint-based methods for cluster identification, our method does not require that clusters be monophyletic nor is it dependent on the selection of a difficult-to-interpret cutpoint parameter. We present an application of our method to data from the Swiss HIV Cohort Study. The method is available as an easy-to-use R package.

## 1. Background

Basic epidemiological models rely on the random mixing assumption [1]. This requires that each individual in a population has an equal probability of coming into contact with any other individual, which can lead to rapid epidemic spread. The random mixing assumption may be appropriate for airborne diseases in small communities. For sexually transmitted infections (STIs) such as HIV-1 however, this hypothesis does not hold: STIs spread within sexual contact networks that limit the propagation to a specific subset of individuals. Identifying the structure of the sexual contact network has multiple applications, for instance, allowing public health officials to target the populations most vulnerable to infection.

Previous studies have shown that the spread of HIV among men who have sex with men is driven by quick transmission chains, i.e. groups of infected individuals with genetically similar viruses [2,3]. We say that patients of such a quick transmission chain form a cluster. A cluster is the result of series of infection events very close in time, and their role in spreading the epidemic affects the efficacy of public health policies: the effectiveness of Treatment as Prevention, a policy currently advocated by the WHO [4], will be limited if most of the transmission happens early after infection, before HIV is diagnosed.

One reason for individuals belonging to a quick transmission chain, i.e. for individuals to form a cluster, is that they are part of the same community in the sexual contact network. A community is defined as a set of nodes in the sexual contact network such that most or all nodes are connected within a community, but few links exist between communities [5]. Communities influence the dynamics of an epidemic: at first, the infection spreads quickly in the community where it has been introduced. The rate of transmission then decreases as the population of susceptibles in the community is progressively exhausted [6]. Eventually, a new introduction event may occur, where an individual from a previously uninfected community is infected through one of the inter-community connections. As the newly infected community is completely susceptible, the rate of transmission then goes up suddenly as new transmission routes open. Thus, the community structure of the sexual contact network shapes transmission dynamics and, in turn, leaves a footprint in the phylogeny reconstructed from pathogen genetic sequences of different infected individuals within an epidemic. In particular, the community structure induces clusters of individuals in the phylogeny.

Previous studies have found varying degrees of influence of the contact network on the phylogeny. Welch [7] found almost no influence of the clustering coefficient of a network on the shape of transmission trees when the degree distribution of the network was kept constant, and Robinson *et al*. [8] found a modest effect of the degree distribution in the network on the shape of phylogenies reconstructed from simulated genetic data. On the other hand, Leventhal *et al*. [9] and Bohme [10] found that the shapes of phylogenies could be significantly affected by variance in degree distribution and mean path length [9] or in degree correlation and clustering coefficient [10] of the contact network. The link between network structures and phylogenies is also affected by viral characteristics such as within-host evolution [11] and recombination [12]. Several methods have been proposed to identify structural characteristics, such as connectivity and clustering coefficient, of the population network from a viral phylogeny [13,14].

Phylogenetic clustering methods aim to find transmission clusters within phylogenies, exploiting the effects that, e.g. contact networks have on phylogenies. To this end, a transmission cluster is defined to be a set of individuals belonging to the same transmission chain within a particular community in the contact network. We do not investigate other reasons for clustering beyond the contact network. Phylogenetic clustering methods, which we will refer to as ‘cutpoint-based’ methods, were evaluated in [15], also under the assumption that clustering is caused by the contact network. These cutpoint-based methods differ in how they define the distance between tips of the tree, but they have two major features in common: first, they require a difficult-to-interpret cutpoint parameter to be specified by the user; second, they assume that the clusters are monophyletic in the phylogeny or monophyletic in a tree obtained from hierarchical clustering (Def. 4 in [15]), i.e. that the most recent common ancestor of all tips belonging to a given cluster has no other descending tips. Villandre *et al*. [15] simulated epidemics and built phylogenetic trees on simulated contact networks, and evaluated the performance of cutpoint-based methods which defined a transmission cluster with cutpoint *x* as either (i) a clade whose tips are separated by a fixed tree distance of at most *x*, where tree distance is the sum of branch lengths, (ii) a clade whose elements are separated by a fixed distance of at most *x*, where distance is the standardized number of different nucleotides between tip sequences, (iii) a clade whose elements are separated by a median pairwise tree distance below *x*, (with *x* an arbitrary percentile of the tree's between-tip distance distribution), where tree distance again corresponds to the sum of branch lengths or (iv) a clade in a dendrogram, i.e. an ultrametric tree obtained from the matrix of between-tip tree distances, cut at height *x*, where the dendrogram was obtained using one of three methods: average linkage, complete linkage or weighted pair-group method of analysis. [15] found that the presence of nested, non-monophyletic, clusters in the tree as well as the choice of the cutpoint have a strong impact on the quality of the recovered clusters. It also established that the cutpoint values giving the best results for cluster recovery are dependent not only on the distance used but also on the structure of the underlying network. Thus, there is a need for a method that does not have these limitations.

Multi-state birth–death (MSBD) models have been widely used to model population structure and analyse phylogenies built from individuals in a structured population [16–19], in phylogeny epidemiological and macroevolutionary applications. Thus, in principle, such a model may be used to study the phylogeny produced by a sexual contact network and to infer which tips in a phylogeny belong to which transmission cluster, with birth rates being transmission rates and death rates being removal rates. The rationale of the inference is to associate each cluster to a state in the MSBD model, with clusters differing in their transmission dynamics through time.

The Binary State Speciation and Extinction [16] and its extension to multiple states MuSSE, included in the package Diversitree [17], were the first efforts to infer state-specific birth and death rates from ultrametric phylogenies, i.e. trees with all tips sampled at the same point in time, where each tip is assigned to a state. In [18], these approaches were extended to non-ultrametric trees. More recently, the Beast2 package BDMM [19] allowed the joint reconstruction of a phylogeny and quantification of the parameters of an underlying MSBD model. These approaches require the user to specify how many states the model contains and to which state each tip of the phylogeny belongs. An exception to the latter is [18], which can integrate over tip states, but does not assign states to tips.

None of the above approaches are directly applicable to the inference of transmission clusters, for two reasons. First, the state of tips, i.e. which cluster they belong to, is not known prior to the analysis. Second, integrating over the tip states instead of explicitly assigning states to tips means that the partition of tips into clusters cannot be inferred.

The method Bayesian analysis of macroevolutionary mixtures [20] addresses these issues and is able to infer the number of states and to assign each tip to a state. Furthermore, the birth- and death-rate parameters associated with each cluster are estimated. However, it was designed to be used with macroevolutionary datasets, meaning at the time of writing it could only analyse ultrametric trees. For epidemiological datasets, we have non-ultrametric trees as samples are collected through time. Furthermore, its results have been called into question, as [21] identified issues regarding the calculation of its likelihood function and its dependency on the user-defined prior for inference of the number of states.

In this paper, we present a new method to identify transmission clusters in a phylogeny built from viral sequences based on the MSBD model. We note that other authors [22] conducted a similar clustering study, relying on pure birth models. McCloskey & Poon [22] highlight in their acknowledgements section that their study builds upon our ideas which we presented at a conference and outline in the following. We assume here that transmission clusters were induced through communities in sexual contact networks. When a pathogen spreads through such a contact network, the transmission rate typically increases upon the pathogen entering a new community, as the number of susceptible individuals in the new community is typically larger. Our method is designed to detect these ‘jumps’ in transmission rate, which we associate with the formation of a new cluster. The method assumes decreasing transmission rates within clusters to account for the depletion of susceptibles. In particular, it does not require prior knowledge on the number of clusters or the tip assignment into clusters. We evaluate the performance of this new method on the simulated dataset of [15] and compare it to cutpoint-based methods. We then apply it to a published HIV phylogeny [14] which was obtained based on 192 sequences from the Swiss HIV Cohort Study (SHCS). Finally, we discuss the limitations of the method and planned future work.

## 2. Methods

### 2.1. Model

We assume an MSBD model similar to the model used in the BDMM package [19]. The birth–death process starts with one infected individual at time *τ* > 0 in the past in an ancestral state (i.e. cluster membership) and is stopped at present time 0. Thus, we measure time in the backward direction, increasing from the present to the root. Without loss of generality, we define the ancestral state to be state 1. State changes occur at a per-lineage rate *γ*. Our MSBD model contains an unknown number of states n*. We assume identical transition rates between clusters, so that the rate *m*_*i*,*j*_ with which hosts in state *i* infect hosts in state *j* is as follows:

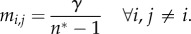


Each individual produces an additional individual at the state- and time-dependent transmission rate *λ*_*i*_(*t*) (function of *λ*_0,*i*_, *z*_*i*_ as defined below), and is removed with a state-dependent removal rate *μ*_*i*_ corresponding to the removal rate or rate of ‘becoming non-infectious’.

The depletion of the susceptible population is modelled by the exponential decay of the transmission rates in the process. Each state is associated with a specific initial transmission rate *λ*_0,*i*_ and a transmission decay rate *z*_*i*_. *λ*_*i*_(*t*) = *λ*_0,*i*_ × e^*z*_*i*_^^(*t*−*t*_0,*i*_^^)^ is the transmission rate of a lineage in state *i* at time *t* before the present, where *t*_0,*i*_ is the time of the first introduction into state *i*. As *t* increases into the past, we impose *z*_*i*_ ≥ 0 so that the transmission rate decreases towards the present.

**Figure 1.**
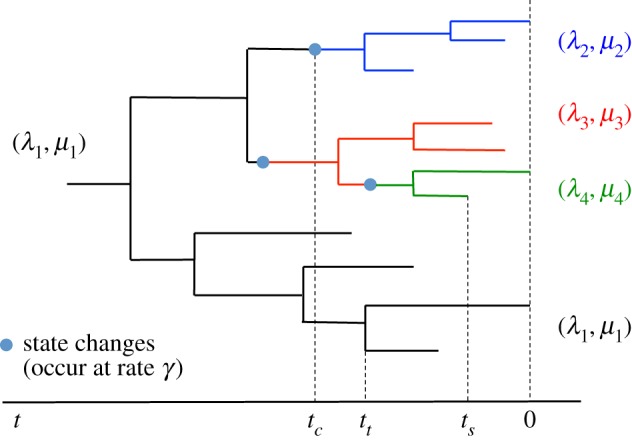
Visual representation of the phylogeny under an MSBD model. Each state is represented by a colour: the ancestral state, in black, starts at the root and represent the first transmission cluster. The other states, in blue, red and green, start at change points along the tree. These states represent further transmission clusters in the course of the epidemic, and the state change points represent the introduction of the pathogen into a new community. Here *t*_c_, *t*_t_ and *t*_s_ are the times of, respectively, a state change, transmission and sampling event, as they appear in equation (2.2).

The infected individuals are sampled upon removal with a probability *σ*. This birth–death model produces a tree on all infected individuals together with position and times of state changes on the tree, and we obtain the phylogeny by considering the subtree spanned by the sampled infected individuals. The phylogeny contains information about the transmission and removal times of the sampled individuals, as well as the positions and times of the state changes, as shown in figure 1. We assume that the state changes correspond to introduction events in newly infected clusters, so that all tips inferred to be in the same state belong to the same transmission cluster.

We refer to a node in the phylogeny being either a branching event, a tip, or a state change event. Edges in the phylogeny connect any two nodes, so any edge belongs to only one state.

### 2.2. Likelihood function

We now derive the probability density of a phylogeny (including the state changes) given the MSBD parameters, i.e. we derive the likelihood of the parameters given a phylogeny with state changes *L*(*M* | *T*): = pdf(*T* | *M*) with pdf being the probability density function. A full derivation of all equations can be found in the electronic supplementary material, text §1.

#### 2.2.1. Differential equations

Following [18,19], the likelihood of the model parameters given the phylogeny can be calculated from the differential equations below. Equation (2.1) describes the probability *p*_*i*_(*t*) of a lineage in state *i* at time *t* not producing any sampled offspring until the present (referred to extinction probability below). Equation (2.2) describes the probability density *q*_*i*,*N*_(*t*) of an edge *N* in state *i* at time *t* evolving according to the phylogeny in time interval [*t*, 0].

2.1
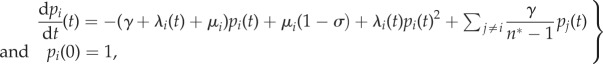

2.2
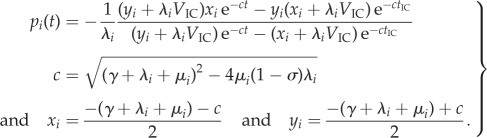


The likelihood of the model parameter given a phylogeny starting at root time *τ* with initial state *I* is *q*_*I*,*N*_(*τ*), meaning the full likelihood can be calculated from equation (2.2). We can also obtain the likelihood per edge by defining the edge likelihood function *f*_*N*_ = *q*_*i*,*N*_(*t*_*b*_)/*q*_*i*,*N*_(*t*_*e*_) for an edge *N* in state *i* with start time *t*_*b*_ and end time *t*_*e*_. *f*_*N*_ follows the differential equation in equation (2.2) with initial condition *f*_*N*_(*t*_*e*_) = 1. The full likelihood of the model *M* given the phylogeny *T* is then obtained by multiplying the likelihoods of all edges as shown in equation (2.3), where *n* is the number of states (including the root state) in the tree, *N*_*i*_ is the set of edges in state *i*, *T*_*i*_ the set of transmission events in state *i* and *S*_*i*_ the set of tips in state *i*.
2.3
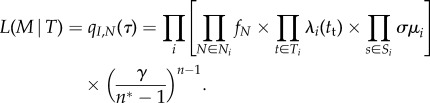


This likelihood function can be applied to trees with or without a root edge, i.e. trees starting with one lineage or two at time *τ*.

### 2.3. Approximations to the likelihood function

The model as described so far is mathematically rigorous; however, performing a maximum-likelihood (ML) inference on this model is computationally complex. As a result, we now introduce several approximations.

#### 2.3.1. Ignoring state changes in unsampled subtrees

The equations for *p* and *f*_*N*_ do not have an analytical solution. Numerical integration is computationally expensive and can be unstable for certain parameters. We thus make the assumption that no state changes happen in the unsampled parts of the tree, meaning all state changes occur on lineages that are observed in the final tree. With this assumption, the master equation for *p*_*i*_(*t*) changes to equation (2.4).
2.4
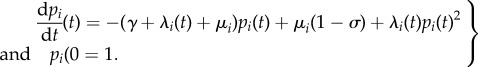


#### 2.3.2. Simplifying the number of states

As the real number of states in the underlying network n* is unknown, we need to estimate it. However, once the approximation described in the previous section is applied, this parameter only appears in the likelihood in the factor (*γ*/(n* −1))^*n*−1^, so maximizing the likelihood is equivalent to minimizing n*. We further assume that each migration enters a previously not visited state, i.e. n* ≥ *n*. Together, the ML estimate will always be n* = *n*. Thus, we fix n* = *n* in the inference.

#### 2.3.3. Time discretization

Equations (2.4) and (2.2) have an analytical solution for constant transmission and removal rates, but not necessarily for time-dependent rates. To obtain a closed form solution, we use time discretization and assume that the transmission rates can be considered locally constant on small enough intervals. The grid size used for the discretization is fixed across the tree and needs to be specified by the user. A smaller size will improve the accuracy of the likelihood calculation but also increase the computational cost.

##### 2.3.3.1. Time discretization for *p*

A closed form of the extinction probability and the likelihood function can be obtained for piecewise constant transmission and removal rates. Assuming constant rates in equation (2.4), and a generic initial condition *p*_*i*_(*t*_IC_) = *V*_IC_ (rather than the initial condition *p*_*i*_(0) = 1), we obtain an analytic solution of equation (2.4)
2.5
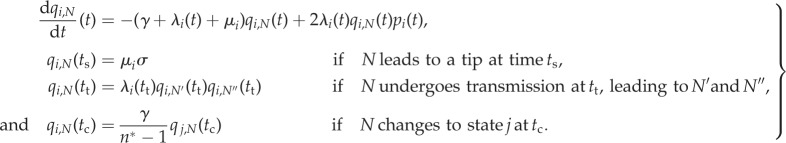
This solution can be verified by differentiating the solution and substituting the result into equation (2.4).

To obtain *p*_*i*_(*t*) using this time discretization, we divide the time interval [*τ*; 0] into a grid. Starting with *p*_*i*_(0) = 1, we can then evaluate *p*_*i*_ using equation (2.5) in each grid interval going backwards in time, using as initial value the solution of the previous grid interval.

##### 2.3.3.2. Time discretization for *f*_*N*_

A closed-form solution of the edge-likelihood function *f*_*N*_ can now be calculated, for a small time interval [*t*_*l*_; *t*_*l*−1_] on an edge *N* in state *i*. This expression uses the value of *p*_*i*_(*t*_*l*−1_), calculated as explained above. We define *f*_*N*_(*t*, *t*_*l*−1_) = *q*_*i*,*N*_(*t*)/*q*_*i*,*N*_(*t*_*l*−1_), and obtain
2.6
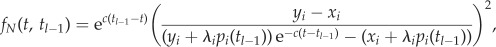
with *c*, *x*_*i*_ and *y*_*i*_ as defined in equation (2.5).

This expression for *f*_*N*_(*t*, *t*_*l*−1_) is a solution of the differential equation (2.2) on the interval [*t*_*l*_, *t*_*l*−1_] with *f*_*N*_(*t*_*l*−1_) = 1, assuming the rates λ_*i*_ are constant in this interval and using the approximate function *p*_*i*_(*t*) from equation (2.5). This can be easily verified by differentiating equation (2.6) and substituting the resulting (d/d*t*)*f*_*N*_(*t*_*l*_, *t*_*l*−1_) into the differential equation (2.2). We then have 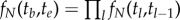.

Equations (2.5) and (2.6) are identical in form to the expressions used in the birth–death skyline model [23], with the piecewise constant birth rate variation being governed by the exponential decay mechanism described earlier.

### 2.4. Algorithm

We now present an algorithm which identifies the cluster configuration and associated parameters that maximize the likelihood in equation (2.3) for a particular phylogeny *T*. More details can be found in the electronic supplementary material, text §2.

#### 2.4.1. Maximum-likelihood search

We use a greedy approach to add state changes until no further improvement of the likelihood can be obtained. New ML estimates are obtained for all transmission, decay, removal and state change rates each time a new state change is added, but the positions and times of previous state changes are fixed.

Once a configuration has been found in which no more state changes can be added to improve the likelihood, we will attempt to recursively remove all the states from this configuration. This step is designed to compensate partly for the fact that the greedy approach never goes back on previous state change assignments, and so can end up in sub-optimal configurations.

Once no further improvements of the likelihood can be obtained by either adding or removing a state, the method will return the best fitting model found, including the state configuration and the ML estimates for all parameters.

The full algorithm is as follows:

(1) Find the most likely parameters for a one-state birth–death model (i.e. with identical birth and death rates across the tree).
(2) For all edges in the tree:
  (a) add a state change on this edge, then
  (b) find the most likely parameters (i.e. transmission, removal and state change rates) for this state configuration, then
  (c) keep this configuration as candidate if its likelihood is higher than previously tested configurations with *n* + 1 states.
(3) If a configuration with *n* + 1 states was found that is more likely than the configuration with *n* states, keep it and go back to step 2.
(4) For each state change in the configuration:
  (a) remove this state change.
  (b) find the most likely parameters for this state configuration, and
  (c) if the configuration without this state was more likely than the previous configuration, keep it.
(5) If at least one state was removed, go back to step 4.
(6) Otherwise, end and record the most likely model.

#### 2.4.2. Time positions of state changes

The model and the likelihood function allow for state changes to be placed anywhere on an edge. However, comparing the likelihood values between configurations with different numbers of states is a problem in an ML framework, as the parameter spaces are of different sizes: indeed, the time of introduction into the *n* + 1th cluster is an additional continuous parameter. To allow an optimization across different numbers of states, we thus limit the positioning of state changes to predetermined discrete positions on edges: they can be positioned at either 10%, 50% or 90% of the length of the edge they are on. An intermediate option is also available, which will test all three predetermined options and keep the most likely.

In the actual epidemic, an introduction event is always simultaneous with a transmission event, which is not possible under our MSBD model. However, cluster introduction events can be placed close to transmission events in the tree, so this should not affect the accuracy of the inference. Furthermore, due to incomplete sampling, the introduction events may actually fall on branches.

### 2.5. Implementation

The likelihood calculation and ML inference are implemented as the publicly available R package ML.MSBD. The package takes as input a phylogenetic tree with branches in units of time, in the phylo format implemented by the package APE [24]. Partial results of the inference are automatically saved after each optimization step, so that an interrupted run can be resumed at any point. The full results returned include the best estimates for the number and positions of cluster introductions, as well as all initial transmission rates, transmission decay rates and removal rates of each state. Furthermore, we return the ML values for each number of states *n* up to 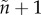 where 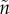 is the ML inferred number of states. An evaluation of the performance of the package can be found in the electronic supplementary material, text §5.

## 3. Results

### 3.1. Cluster inference on simulated data

#### 3.1.1. Dataset

We use a simulated dataset produced by Villandre *et al*. [15], which contains simulated epidemics on three different types of networks, A, B and C. The network structure A is composed of 13 communities of 20 subjects each, with each community being a fully connected graph and one bridge linking any two communities.

The network structure B consists of one central community of size 60, representing a main sexual contact network, connected by single bridges to 25 communities of size 20. Each small community is a fully connected graph. These small communities represent disjoint sexual contact subnetworks in a population of interest.

The network structure C contains 100 communities, whose size was sampled from a distribution obtained from a phylogeny of the SHCS dataset (see [15] for details). To ensure that all communities are accessible, they are first linked in a chain. Additional bridges are then created by connecting any two vertices belonging to different communities with probability 0.00075.

In all network types, edges between communities are weighted with a weight of 0.25, 0.5, 0.75 or 1, meaning that the rate of transmission on these edges is respectively 25%, 50%, 75% and 100% of the transmission rate on within-community edges.

Epidemics were simulated on these networks starting from one random introduction in A networks, one random introduction in the main community in B networks, and two random introductions in C networks. All infected individuals were sampled upon removal and a transmission tree was built from the sampled tips. Thus, there is no phylogenetic uncertainty in this dataset: the tree represents exactly the simulated epidemic. For each type of network (A,B,C) and each weighting scheme (*w* = 0.25, 0.5, 0.75 or 1), 300 epidemics were simulated, for a total dataset of 3600 trees.

Network structure B was designed to correspond best to the monophyletic assumption of the cutpoint-based clustering methods: the epidemic starts in the main cluster and the smaller communities are not connected with each other, so all infections originating from the same introduction will be grouped in a single clade. Network structure A, on the other hand, allows for the possibility of multiple introductions in the same community and onward transmission in further communities inducing nested clusters, thus breaking some of the assumptions of the cutpoint-based methods. More details about the networks are presented in the electronic supplementary material, text §3.

#### 3.1.2. Comparison of our method with cutpoint-based methods

We ran our ML inference on the trees to assign states to tips, and interpret different states as different transmission clusters. In accordance with the simulation conditions, we set *σ* = 1 in the inference. The removal rates *μ*_*i*_ are assumed independent of the community, and so were set to the same value *μ* for all states. The time positions of the state changes were fixed using the intermediate option of testing positions at 10%, 50% and 90% of the length of the edges.

The correspondence between the real network communities and the clusters inferred from the tree was assessed using the adjusted Rand index (ARI) [25,26]. This index measures the number of pairs of tips which are clustered identically in both clusterings, i.e. either in the same cluster or in two different clusters in both arrangements, compared to the number of pairs which are clustered differently. The ARI ranges from −1 to 1: a value of 1 indicates a perfect match of the inferred clustering to the truth, a value of 0 indicates that the inferred clustering matches the truth no better than a clustering drawn at random, and negative values indicate that the inferred clustering is worse than would be expected by chance.

We compare the results from our method to the results obtained by Villandre *et al*. [15] using cutpoint-based clustering methods.

**Figure 2.**
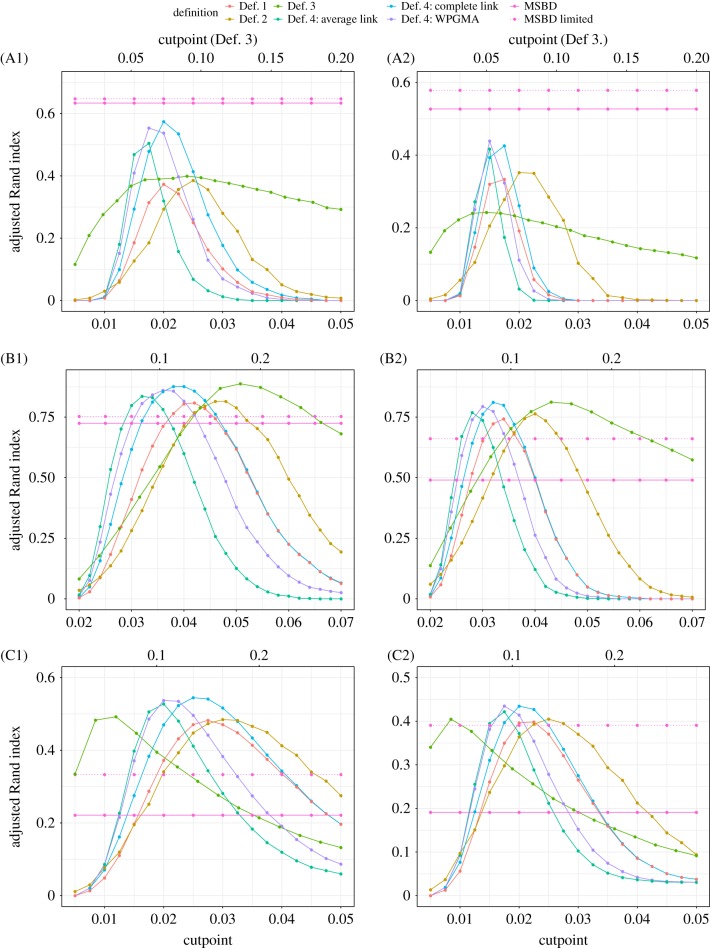
Comparison of the average ARI obtained by the different clustering methods in function of the cutpoint on networks A (parts A1,A2), B (parts B1,B2) and C (parts C1,C2). For each network, the first column (part 1) shows the results for weight *w* = 0.25 and the second column (part 2) for *w* = 1. Our proposed MSBD method is not dependent on a cutpoint. The cutpoint for Definition 3. is shown on the top *x*-axis, the cutpoints for all other definitions are shown on the bottom *x*-axis.

Figure 2 and electronic supplementary material, figure S2 show the scores obtained by our MSBD method on the simulated A,B,C networks compared to the scores of the cutpoint-based clustering methods, respectively, for weights *w* = 0.25 and *w* = 1 and for weights *w* = 0.5 and *w* = 0.75. All methods used the same cutpoints values, except for the method based on Definition 3 (Def. 3). Data corresponding to this method were rescaled to fit in the same figure.

As shown in [15], the results of the cutpoint-based methods are highly variable and good scores can only be obtained from a narrow range of cutpoints. In addition, the best cutpoint value is highly dependent on the underlying network structure: in methods other than Def. 3, the best scores are obtained for a cutpoint of *c* ≈ 0.15 for networks A, *c* ≈ 0.03 for networks B and *c* ≈ 0.02 for networks C. For Def. 3, the best score is obtained for *c* ≈ 0.05 for networks A, *c* ≈ 0.16 for networks B and *c* ≈ 0.04 for networks C. We define the ‘peak range’ of cutpoints for each method, network structure and weighting scheme as the range of cutpoints which give a score that is at least 75% of the best score obtained for any cutpoint. With this definition, the peak ranges are very narrow, with an average length of, respectively, 0.008, 0.015 and 0.016 for networks A, B and C in methods other than Def. 3. The peak ranges obtained with Def. 3 are much wider, but a direct comparison is difficult due to the different definition used for the cutpoint. In all methods, the peak ranges for networks A and C on the one hand, and B on the other hand have very little overlap and the best cutpoint for B is never found in the peak range of either A or C, and vice versa. In conclusion, it is impossible to get good results from all network types with any single cutpoint value.

In addition, the cutpoint-based methods are sensitive to network features and in particular limited by the monophyletic assumption. In both the A and C networks, the best score obtained by any cutpoint-based method is ≈ 0.45 for the weighting scheme *w* = 0.25 and ≈ 0.55 for *w* = 1, whereas it goes up to ≈ 0.85 and ≈ 0.9, respectively, in networks B. An example of inference on a tree with nested cluster introductions is shown in electronic supplementary material, figure S3.

In comparison, the MSBD method performs less well on B networks, with an average score of 0.73 for *w* = 0.25 and 0.49 for *w* = 1. However, it performs much better on A networks, with an average score of 0.64 for *w* = 0.25 and 0.53 for *w* = 1. The worst results are obtained on the C networks, where the average score is ≈ 0.2 for all weights, less than half the best scores obtained by cutpoint-based methods.

The low scores obtained on the C networks point to a potential limitation of our method on the number of clusters that can be inferred from a tree. The trees simulated on the C networks contain clusters that have on average fewer elements and a higher proportion of very small clusters than the trees simulated on the A and B networks. These clusters may be harder to detect due to their low signal. This is supported by the number of inferred clusters shown in electronic supplementary material, table S1, which shows that the MSBD inference infers the correct number of clusters for networks A and B, but strongly underestimates it in networks C. To confirm this hypothesis, we calculated the scores obtained by the MSBD method when excluding all tips that belonged to a cluster with strictly less than eight tips. The results are shown in figure 2 (dotted line). The proportion of tips excluded by applying this criterion is shown in table 1. The scores of all network structures and all weighting schemes improved when applying this criterion. The improvement increased with the proportion of tips belonging to the excluded clusters, supporting our hypothesis that the MSBD method has difficulty identifying them. In particular, the MSBD scores on the C network structure for weight ≥0.5 increase to a level on par with the best scores obtained by cutpoint-based methods.

**Table 1.** Percentage (%) of tips belonging to clusters with strictly less than eight tips, per network structure and weighting scheme.

network type	A	B	C
*w* = 0.25	9.0	9.9	33.9
*w* = 0.5	15.3	16.0	44.6
*w* = 0.75	21.7	20.8	51.4
*w* = 1	25.1	22.2	55.5

### 3.2. Quality of the parameter inference

To evaluate the performance of our MSBD method beyond cluster identification, we simulated several datasets of 200 trees each under the MSBD process, with various parameter combinations. Simulations were done using Gillespie's algorithm [27] for forward simulation of stochastic processes. Birth–death trees were simulated to have either one state (*γ* = 0) or multiple states sharing the same birth, birth decay and death rates (*γ* > 0). Tips were sampled upon removal and the process was run until the tree reached 50 sampled tips. The MSBD method was then applied directly to the simulated trees. Because these trees were not built from network simulations, we did not try to assess the quality of the cluster inference, but we focused on the quality of the parameter inference and on whether our method can adequately distinguish between trees that contain several states and trees that do not.

The results are summarized in table 2. We can see that although the MSBD method is able to consistently infer multiple states when they are present, it will also wrongly detect one additional state in around 25% of the trees that only contain one state. This may be a problem of noise, where due to the stochasticity of the simulation one subtree is slightly more likely when attributed different rates than the rest of the tree. This problem can be alleviated by looking at the difference in the inferred transmission rates of each state, which are also estimated by our method: a smaller difference is more likely to be indicative of noise. As previously noted, the method also tends to underestimate the number of states in multi-state trees, mostly because it cannot detect states with only a few tips.

**Table 2.** Parameter inference on simulated datasets. Each dataset contains 200 trees of 50 tips each, simulated under an MSBD process using Gillespie's algorithm. Transmission rates are averaged over the entire tree.

dataset parameters		*λ*_0_ = 25, *z* = 12, *μ* = 1, *γ* = 0	*λ*_0_ = 25, *z* = 15, *μ* = 1, *γ* = 0	*λ*_0_ = 10, *z* = 1, *μ* = 5, *γ* = 0.5	*λ*_0_ = 10, *z* = 2, *μ* = 5, *γ* = 0.5
average number of clusters	simulated	1	1	4.95	6.38
	>5 individuals, simulated	1	1	1.92	2.49
	inferred	1.22	1.25	2.43	2.65
average transmission rate	simulated	1.09	0.86	6.95	5.40
	inferred	1.54	1.38	7.52	6.20
	median absolute error	0.37	0.49	0.75	0.78
average removal rate	simulated	1.0	1.0	5.0	5.0
	inferred	0.88	0.91	4.64	4.50
	median absolute error	0.21	0.20	0.73	0.71

Regarding the parameter inference, the method has a slight bias towards overestimating the transmission rate and underestimating the removal rate. This is potentially due to our simulation process being conditioned on reaching 50 tips, which could bias datasets in favour of trees showing apparent higher diversification rates [28]. Overall, the absolute error on the inferred parameters remains low compared to the true values, both in datasets with one cluster and in datasets with multiple clusters.

We performed a similar analysis on datasets simulated with incomplete sampling *σ* = 0.75 or *σ* = 0.5, shown in electronic supplementary material, tables S4 and S5 (electronic supplementary material, text §6). The accuracy of the parameter inferences decreases with lower sampling proportions, in particular in the transmission rate estimates. However, the relative error remains low and the MSBD parameter estimates remain reliable even with lower sampling proportions.

In conclusion, the parameter inference from the MSBD method is reliable, although it suffers from noise when applied to trees which contain only one state.

### 3.3. Cluster inference on HIV dataset

In this section, we analyse a tree used in another study of the correlation between sexual networks and tree features [14]. HIV-1 subtype B pol sequences were obtained from the SHCS 192. While the Swiss epidemic includes a mixture of population risk groups including heterosexuals, injecting drug users and MSM, only viral samples from MSM were analysed. A large cluster including almost 200 sampled individuals who predominantly lived or sought treatment in the Zürich area was identified from an ML phylogeny of the complete dataset. The phylogeny of this cluster was then obtained by fitting a SIR-type pairwise epidemic model to this sub-epidemic while simultaneously inferring the tree from the sequence data in BEAST2. We re-analyse the maximum clade credibility tree provided for that cluster in the supporting information of [14].

**Figure 3.**
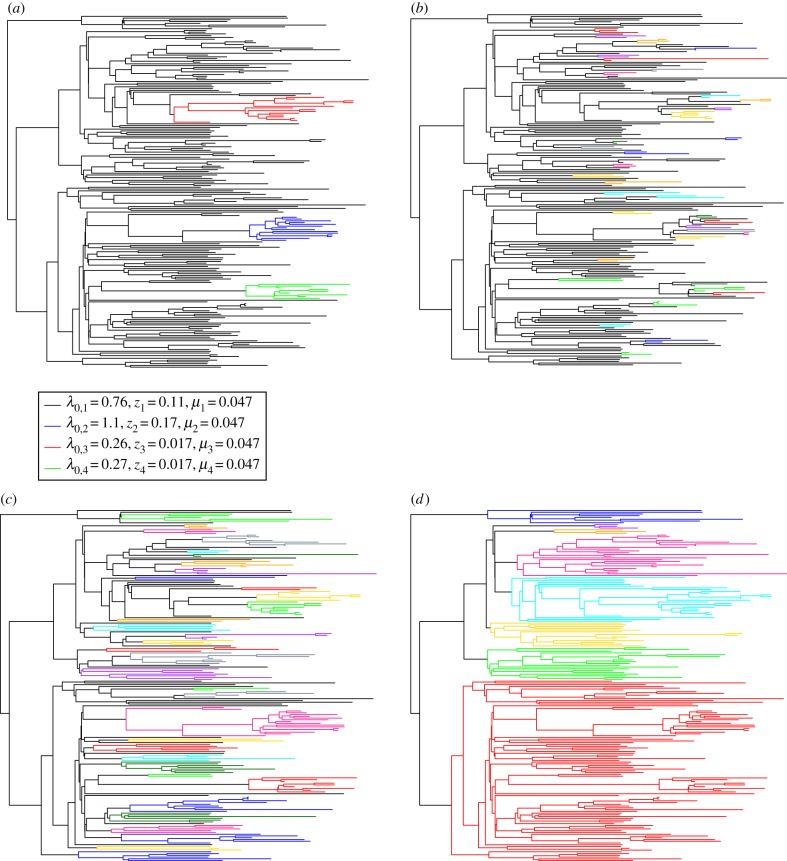
Analysis of the empirical HIV tree. Comparison of the clusters obtained with MSBD (*a*) or with Cluster Picker with a bootstrap threshold of 0.0 and a genetic distance threshold of 1.5% (*b*), 4.5% (*c*) and 8% (*d*).

The results of the MSBD analysis are shown in figure 3*a*. Three sub-clusters are identified in the tree, one with a higher base transmission rate than in the backbone of the tree, and two with similar base transmission rates which are lower than in the backbone of the tree.

We compare our results to results obtained using the software Cluster Picker [29] and PhyloPart [30], phylogenetic cutpoint-based method which detect clusters based on a combination of bootstrap support at the nodes and respectively genetic distance between tip sequences or patristic distances. Genetic sequences were generated for the tree using the software SeqGen [31] (see electronic supplementary material, text §4 for more details).

The results of Cluster Picker are shown in figure 3 and the results of PhyloPart in electronic supplementary material, figure S4. As both led to similar results, we will only discuss the results for Cluster Picker here. As with other cutpoint-based methods, the results depend strongly on the user-defined values. We used three different cutpoint values for the genetic distance: 1.5%, 4.5% and 8%. 4.5% is the default value proposed by Cluster Picker and is the higher bound of the range recommended by Cluster Picker for HIV data, whereas 1.5% is the lower bound of the recommended range. For the bootstrap support threshold, we used the value 0.0. With this value, the bootstrap support is disregarded entirely, which mimics the behaviour of the methods studied by [15]. The results are shown in figure 3. We observe that the number of identified clusters is strongly dependent on the cutpoint values, in keeping with the results obtained by [15]. The size of the identified clusters varies also widely, even within the bounds of the recommended range of cutpoints.

One interesting thing to note is that the cluster pattern identified by MSBD cannot be obtained by Cluster Picker even when varying the threshold used. Cutpoint thresholds which include all of the (non-root) clusters detected by MSBD typically include additional small clusters which are not supported by the MSBD model. On the other hand, reducing the threshold to avoid these spurious clusters causes Cluster Picker to miss clusters supported by MSBD.

## 4. Discussion

We have introduced a novel method of identifying transmission clusters from a phylogeny, based on an MSBD model. This model is designed to identify transmission clusters induced by a pathogen spreading in a contact network with communities. Transmission clusters are defined as all individuals belonging to a transmission chain within a single community. It will be an interesting future work to investigate the ability of the MSBD model to infer clusters formed due to other dynamics such as superspreading. Beyond HIV, our method is applicable to any epidemic where the transmission dynamics are governed by a contact network structure.

Our likelihood function makes two important assumptions: the first one is that each community is entered precisely once, and the second one is that unsampled subtrees, i.e. subtrees that do not appear in the reconstructed phylogeny, do not contain cluster introduction events. The implementation also relies on a time discretization which approximates all transmission rates as locally constant on small time intervals. A similar discretization can be applied to extend our method to time-dependent removal rates, which are not currently supported.

This new method has a few key differences compared to the cutpoint-based clustering methods. Firstly, it is not restricted to monophyletic clades and can thus find clusters that are nested within one another in the phylogeny. As a result, our method clearly outperformed the others on simulated networks which were designed specifically to violate the monophyletic assumption. This issue is particularly problematic in datasets that have been sampled over an extended period of time, as nested introduction events are more likely to appear there.

Secondly, as the MSBD method is model-based, it does not rely on an arbitrary cutpoint to be chosen by the user. Instead, we look for significant changes in transmission rates. While our method does involve approximations, our simulation studies show that these do not destroy the statistical signal for cluster detection. The use of a likelihood function also gives an estimate of the statistical significance of each detected cluster and allows us to easily score and compare different cluster partitions. Moreover, as the parameters of the model are biologically meaningful, they are therefore easier to potentially fix or interpret in an objective way.

The intuitive reason for these two key differences of MSBD to cutpoint-based methods is that the MSBD method looks at relative changes in branch lengths, while cutpoint-based methods cluster individuals up to a particular distance in absolute branch lengths. Thus, the cutpoint-based methods only find monophyletic clusters. Furthermore, branch lengths on a different scale (e.g. all distances are multiplied by 100) require a different cutpoint. On the other hand, the MSBD method uses likelihood statistics to determine if the relative branch length changes are significant, indicating a new cluster.

Avoidance of a cutpoint parameter is an important advantage. Villandre *et al.* [15] showed that the quality of the detection achieved by phylogenetic cutpoint-based clustering methods is highly sensitive to the value of this parameter, regardless of underlying contact network type. In particular, they showed that it is impossible to define a single cutpoint value as adequate for all network types, even for the same pathogen. Cluster Picker recommends cutpoints between 0.015 and 0.045 for HIV based on previous empirical studies of HIV. However, our results show that very different cluster partitions can be obtained at the two ends of this range. Cutpoint values will further be impacted by the evolutionary rate and other epidemiological dynamics, meaning these guidelines cannot be easily transferred to another pathogen. The chosen cutpoint value is strongly linked with the number of clusters inferred by cutpoint-based methods, thus obtaining the correct clusters requires prior knowledge of the true number of clusters.

Overall, while our method may not perform as well on certain types of network as phylogenetic cutpoint-based methods were we to know the optimal cutpoint in advance, it outperforms the cutpoint-based methods on a wide range of cutpoints on simulated datasets following our assumptions on pathogen spread. Furthermore, while there exist other non-phylogenetic clustering methods, such as HIV-TRACE [32], which can detect non-monophyletic clusters, these methods still require specification of a cutpoint parameter and we expect that they suffer from the same issues as the phylogenetic cutpoint-based methods.

MSBD methods have a strong limitation on the size of clusters that can be inferred from a tree. This is seen from the low scores obtained on the fragmented type C networks and the improvements obtained when only investigating if clusters of a certain size are inferred. Contrary to the cutpoint-based methods, which can handle arbitrary numbers and sizes of clusters, our method can only add clusters when there is a strong signal for them and thus performs worse than cutpoint-based methods in datasets with many small clusters. Again, this comparison relies on knowing the optimal cutpoint, and as said above, this knowledge is typically not available.

Another limitation of the current implementation is its computational cost, which limits the size of the trees that can be analysed. (Current run time is on the order of CPU-days for a few hundred tips.) In contrast to cutpoint-based methods, the MSBD method thus cannot handle datasets containing tens of thousands of sequences. Future work will focus on implementing the algorithm in parallel and exploring other possible approximations, with the aim of increasing speed without lowering precision. Making use of the insight that the MSBD method employs the information on changes in relative branch lengths may be a future guide to fast but accurate methods.

In the future, we plan to implement our model in a Bayesian framework, which will allow us to explore the number and positions of clusters in a more rigorous way by using reversible jumps instead of multiple rounds of optimization. Some approximations could be removed in this framework, such as the assumption that *n* = n* or the fixed positions of state changes on edges. Moreover, estimating the uncertainty around the various estimated parameters is problematic in an ML framework, in particular for the positions and number of state changes.

This is compounded by the fact that the current method uses a timed phylogeny as input, and thus relies strongly on this phylogeny being inferred correctly. This also means that the uncertainty associated with the phylogenetic inference cannot be integrated in the cluster inference. Again, a Bayesian framework could solve this issue by allowing a joint inference of the phylogeny and the clusters, using the MSBD as a model-based prior on the transmission tree.

In summary, in spite of the limitations discussed, our results clearly show that an approach based on a statistical model for cluster detection can overcome some important deficiencies of previous approaches. In particular, the assumption of clusters being monophyletic can be dropped, and the sensitivity to non-biological cutpoint parameters can be completely avoided.

## Supplementary Material

Supplementary text

## Supplementary Material

Code files

## Supplementary Material

Data files

## Data accessibility

All analyses, pre- and post-processing of the datasets were done using custom R scripts. These scripts and the datasets are included in the electronic supplementary material.
