# Supplementary material for: Detection of HIV transmission clusters from phylogenetic trees using a multi-state birth–death model
Source: J R Soc Interface. 2018 Sep 5;15(146):20180512. doi: 10.1098/rsif.2018.0512 (PMC6170769; doi:10.1098/rsif.2018.0512)
Supplement: Supplementary text [file rsif20180512supp1.pdf]

Detection of HIV transmission clusters from phylogenetic trees using a multi-states birth-death model
Supplementary Materials

1 Derivation of the likelihood

We show here how the equations involved in the calculation of the MSBD likelihood are derived.

1.1 Variables involved

- λ_i birth (transmission) rate for state i, dependent on time such that $\lambda_i(t) = \lambda_{0,i} \times e^{z_i(t-t_{0,i})}$
- μ_i death (removal) rate for state i
- γ state change rate
- $m_{i,j} = \frac{\gamma}{n^*-1}$ rate of transition from state i to state j
- $p_i(t)$ the probability of a lineage in state i at time t not appearing in the phylogeny, i.e going extinct before the present and/or not being sampled
- $q_{i,N}(t)$ the probability of an edge N in state i at time t evolving until the present as shown in the phylogeny
- $f_N = \frac{q_{i,N}(t_b)}{q_{i,N}(t_e)}$ the likelihood of an edge N in state i which starts at time t_b and ends at time t_e

1.2 Master equations

Since all processes involved (birth, death and state changes) are Poisson processes, they have exponential waiting times, and we can list all possible events which can happen on a lineage in state i between time $t + \delta t$ and t:

- a birth happens with probability $\lambda_i \delta t + o(\delta t)$
- a death happens with probability $\mu_i \delta t + o(\delta t)$
- a state change to the new state j happens with probability $\frac{\gamma}{n^*-1} \delta t + o(\delta t)$
- multiple events happen with probability $o(\delta t)$
- no event happen with probability $1 - (\lambda_i + \mu_i + \gamma)\delta t + o(\delta t)$

This allows us to write

$$p_i(t+\delta t) = (1-(\lambda_i+\mu_i+\gamma)\delta t)p_i(t) + \lambda_i \delta t p_i(t)^2 + \mu_i \delta t(1-\sigma) + \sum_j \frac{\gamma}{n^*-1}\delta t p_j(t) + o(\delta t)$$

leading to the master equation

$$\frac{dp_i}{dt}(t) = -(\gamma+\lambda_i(t)+\mu_i)p_i(t) + \mu_i(1-\sigma) + \lambda_i(t)p_i(t)^2 + \sum_{j\neq i}\frac{\gamma}{n^*-1}p_j(t)$$

Similarly we have

$$q_{i,N}(t+\delta t) = (1-(\lambda_i+\mu_i+\gamma)\delta t)q_{i,N}(t) + 2\lambda_i \delta t p_i(t) q_{i,N}(t) + o(\delta t)$$

leading to the second master equation

$$\frac{dq_{i,N}}{dt}(t) = -(\gamma+\lambda_i(t)+\mu_i)q_{i,N}(t) + 2\lambda_i(t)q_{i,N}(t)p_i(t)$$

1.3 Solving the approximate equation for p_i

As explained in the main text, we now want to solve the approximate equation

$$\frac{dp_i}{dt}(t) = -(\gamma+\lambda_i(t)+\mu_i)p_i(t) + \mu_i(1-\sigma) + \lambda_i(t)p_i(t)^2$$

with the initial condition $p_i(t_{IC}) = V_{IC}$ and assuming that $\lambda_i(t) \approx \lambda_i$ on the interval $[t_{IC}, t]$. Let $v_i(t) = \lambda_i p_i(t)$, we obtain

$$\frac{dv_i}{dt} = -(\gamma+\lambda_i+\mu_i)v_i(t) + \mu_i(1-\sigma)\lambda_i + v_i(t)^2$$

Let $v_i(t) = -\frac{u_i'(t)}{u_i(t)}$, the equation becomes

$$u_i''(t) + (\gamma+\lambda_i+\mu_i)u_i'(t) + \mu_i(1-\sigma)\lambda_i u_i(t) = 0$$

The solutions are given by solving

$$x^2 + (\gamma+\lambda_i+\mu_i)x + \mu_i(1-\sigma)\lambda_i = 0$$

$\Delta = (\gamma+\lambda_i+\mu_i)^2 - 4\mu_i(1-\sigma)\lambda_i \geq (\gamma+\lambda_i+\mu_i)^2 - 4\mu_i\lambda_i = \gamma^2 + 2\gamma(\lambda_i+\mu_i) + (\lambda_i-\mu_i)^2 \geq 0$

Let $c = \sqrt{\Delta}$, we get solutions

$$x_i = \frac{-(\gamma+\lambda_i+\mu_i)-c}{2} \quad \textit{and} \quad y_i = \frac{-(\gamma+\lambda_i+\mu_i)+c}{2}$$

Then, with A and B arbitrary constants, we obtain

$$u_i(t) = Ae^{x_i t} + Be^{y_i t}$$

$$u_i'(t) = Ax_i e^{x_i t} + By_i e^{y_i t}$$

Thus

$$p_i(t) = -\frac{u_i'}{\lambda_i u_i} = -\frac{1}{\lambda_i}\frac{Ax_i e^{x_i t} + By_i e^{y_i t}}{Ae^{x_i t} + Be^{y_i t}} = -\frac{1}{\lambda_i}\frac{Ax_i e^{-ct} + By_i}{Ae^{-ct} + B}$$

From initial condition $p_i(t_{IC}) = V_{IC}$,

$$Ax_i e^{-ct_{IC}} + By_i = -(\lambda_i V_{IC})(Ae^{-ct_{IC}} + B)$$

$$A(x_i + \lambda_i V_{IC})e^{-ct_{IC}} = -B(\lambda_i V_{IC} + y_i)$$

Finally

$$\begin{aligned} p_i(t) &= -\frac{1}{\lambda_i}\frac{A(y_i + \lambda_i V_{IC})x_i e^{-ct} - y_i B(\lambda_i V_{IC} + y_i)}{A(y_i + \lambda_i V_{IC})e^{-ct} - B(\lambda_i V_{IC} + y_i)} \\ &= -\frac{1}{\lambda_i}\frac{(y_i + \lambda_i V_{IC})x_i e^{-ct} - y_i(x_i + \lambda_i V_{IC})e^{-ct_{IC}}}{(y_i + \lambda_i V_{IC})e^{-ct} - (x_i + \lambda_i V_{IC})e^{-ct_{IC}}} \end{aligned}$$

1.4 Solving the approximate equation for f_N

The edge likelihood function f_N fulfils the equation

$$\frac{df_N}{dt}(t) = -(\gamma + \lambda_i(t) + \mu_i)f_N(t) + 2\lambda_i(t)f_N(t)p_i(t)$$

on the interval $[t_b; t_e]$, with the initial condition $f_N(t_e) = 1$ where t_b is the start of the edge N and t_e the end of the edge.

Similarly to the previous section, we assume that λ_i is locally constant on the interval $[t; t_e]$. We will use the equation for $p_i(t)$ based on the initial condition $p_i(t_e) = V_{IC}$. The solution is given by

$$f_N = \frac{C_N}{u_N(t)}$$

where

$$\begin{aligned} u_N(t) &= exp(\int [-2\lambda_i p_i(t) + (\gamma + \lambda_i + \mu_i)]dt) \\ &= exp(2ln(u_i(t)) + (\gamma + \lambda_i + \mu_i)t) \\ &= u_i(t)^2 e^{(\gamma+\lambda_i+\mu_i)t} \end{aligned}$$

Thus

$$\begin{aligned} f_N(t) &= \frac{C_N}{u_i(t)^2 e^{(\gamma+\lambda_i+\mu_i)t}} = \frac{C_N}{(Ae^{x_i t} + Be^{y_i t})^2 e^{(\gamma+\lambda_i+\mu_i)t}} \\ &= \frac{C_N}{(Ae^{-ct} + B)^2 e^{ct}} = \frac{(\lambda_i V_{IC} + y_i)^2 C_N}{(A(\lambda_i V_{IC} + y_i)e^{-ct} - A(x_i + \lambda_i V_{IC})e^{-ct_e})^2 e^{ct}} \\ &= \frac{(\lambda_i p_i(t_e) + y_i)^2 C'_N e^{-ct}}{((\lambda_i p_i(t_e) + y_i)e^{-ct} - (x_i + \lambda_i p_i(t_e))e^{-ct_e})^2} \end{aligned}$$

The initial condition $f_N(t_e) = 1$ gives

$$\begin{aligned} (\lambda_i p_i(t_e) + y_i)^2 C'_N e^{-ct_e} &= ((\lambda_i p_i(t_e) + y_i)e^{-ct_e} - (x_i + \lambda_i p_i(t_e))e^{-ct_e})^2 \\ &= e^{-2ct_e}(y_i - x_i)^2 \end{aligned}$$

Thus $(\lambda_i p_i(t_e) + y_i)^2 C'_N = e^{-ct_e}(y_i - x_i)^2$.

Finally we obtain

$$\begin{aligned} f_N(t) &= e^{-c(t_e+t)} \left(\frac{y_i - x_i}{(y_i + \lambda_i p_i(t_e))e^{-ct} - (x_i + \lambda_i p_i(t_e))e^{-ct_e}} \right)^2 \\ &= e^{c(t_e-t)} \left(\frac{y_i - x_i}{(y_i + \lambda_i p_i(t_e))e^{-c(t-t_e)} - (x_i + \lambda_i p_i(t_e))} \right)^2 \end{aligned}$$

2 Details on the algorithm

2.1 Detailed time discretization

Here we describe how to calculate f_N and obtain an evaluation of the likelihood provided in Eq. 4, using Eq. 7. Values of p_i for all branching times and state change times are precomputed to avoid the repetition of those calculations for multiple edges. For edge N in state i starting at time t_b and ending at time t_e (i.e. $t_b < t_e$), we aim to calculate $f_N(t_b, t_e)$. Thus we aim to solve, using the time discretization, the differential equation 3 with initial value $f(t_e, t_e) = 1$:

1. Fetch the precomputed value of $p_i(t_e)$.
2. Divide the interval $[t_b, t_e]$ in k equidistant intervals $[t_k, t_{k-1}], [t_{k-1}, t_{k-2}], \ldots, [t_1, t_0]$ with $t_0 = t_e$ and $t_k = t_b$.
3. For each step $l \in [1..k]$ do the following:
   (a) calculate $\lambda_{i,l}$ the mean of $\lambda_i(t)$ on the interval $[t_l, t_{l-1}]$, then
   (b) calculate $p_i(t_l)$ and $f_N(t_l, t_{l-1})$ by using the constant rates solutions provided in Eq. 6 for p and in Eq. 7 for f with $\lambda_i = \lambda_{i,l}$, based on the value $p_i(t_{l-1})$ given by the precomputed value if $l = 1$ and by the previous step $l - 1$ otherwise.
4. Finally, compute $f_N(t_b, t_e) = \prod_{l=1}^{k} f_N(t_l, t_{l-1})$.

2.2 Algorithm: initial condition

The first step of the algorithm is to infer the most likely parameters for a constant birth-death model, i.e for a model with only one state, given the tree. These parameters will be used as starting values for the optimization in further steps, to minimize the impact of user-provided initial values. The initial values used in this initial optimization can have a great impact on the entire inference: if they are too distant from the maximum likelihood estimates (MLEs), it can happen that the one-state optimization finds only a local optimum of the parameter values, and this will in turn affect all subsequent steps of the inference. Our method avoids this issue by applying an initial coarse-grained optimization step prior to the main optimization algorithm. Initial values are tested until no further improvement of the likelihood in the one-state configuration can be obtained. The MLEs obtained will then be used to initialize all further steps of the algorithm.

3 Features of simulated networks A, B and C

Various features of the A,B,C networks and the resulting simulated trees are shown in table 1. Networks A and B are very similar both in the size of their trees and in the cluster partition inside trees. Network C, on the other hand, contains a large number of fairly small clusters.

Even though C trees are much larger on average, the clusters they contain are very small on average and 34% of them include only 1 or 2 tips of the tree. These very small clusters contain very little signal from the underlying contact network, and thus are not expected to be detected by the method.

Network type	A	B	C
Number of clusters in the network	13	26	100
Number of elements per cluster	20	21.5	9.8
Number of tips per tree	52	60	196
Number of clusters per tree	6.0	6.6	39.1
Inferred number of clusters	6.6	5.3	16.4
Number of elements per cluster in the tree	9.5	9.6	5.2
Proportion of small clusters (<3 elements) in trees	21%	14%	34%

Table 1: General features of the A, B, C networks. All numbers are averages over the 4 weighting schemes, i.e averages over all 1200 trees in each network.

4 Sequence simulation on HIV empirical tree

Sequences were generated on the HIV empirical tree using a GTR model with a gamma distribution with 4 rate categories and invariant sites. The parameters of the molecular evolution model were set to the estimates obtained by (1) when inferring the tree, which are shown in table 2.

5 Performance of the MSBD inference

5.1 Speed improvement option

The algorithm as presented in the main text is fast at the beginning of the inference but will progressively slow down as more states are added, due to the increase in the number of parameters that need to be optimized.

We have thus added a so-called 'fast optimization' option, which limits the number of parameters which are allowed to change during one step of the maximum likelihood optimization. In practice, when adding the n-th state change, only the parameters $\lambda_{0,n+1}$, $\lambda_{0,a}$, z_{n+1}, z_a, μ_{n+1} and μ_a are optimized, where a is the state from which the epidemic is introduced in state $n+1$. All other parameters are fixed to the values inferred with n states. Thus this option results in each step of the algorithm having a constant cost instead of a cost dependent on n, however some precision is lost by fixing parameters.

It is also possible to run the normal analysis for the early steps of the algorithm and turn on the fast optimization afterwards.

5.2 Speed evaluation

We measured the CPU time necessary to run the MSBD inference on trees from networks A, B and C on a single core of the Euler cluster of the ETH Zürich, shown in Table 3. Inferences on

Parameter	Value used
Proportion of invariant sites	48%
Frequency of A	0.38
Frequency of C	0.16
Frequency of G	0.22
Frequency of T	0.24
Shape of gamma heterogeneity	0.57
Substitution rate	0.0015
Transition rate $A \to C$	0.23
Transition rate $A \to G$	1.12
Transition rate $A \to T$	0.09
Transition rate $C \to G$	0.14
Transition rate $C \to T$	1.0
Transition rate $G \to T$	0.11

Table 2: Parameter values used to simulate sequences with SeqGen.

the A and B networks took a few days of CPU time, however the inference on C networks was much slower and had to be completed using the "fast optimization" option.

Network	A	B	C	C (with fast option)
Average CPU time (s)	130000	78562	> 1025409	468089
Average CPU time (days)	1.5	0.9	> 11.9	5.4

Table 3: Average CPU time of the MSBD inference. The inference was performed with the regular optimization on networks A and B, and on C with the fast optimization. Inferences on the C networks with the regular optimization were stopped after 12 days of CPU time.

We also compared directly the performance of the "fast optimization" option and the regular algorithm, using a dataset of 300 trees of average size 200 tips. We first performed a partial inference on this dataset, which was stopped once either 5 or 15 state changes had been inferred on each tree. The algorithm was then restarted from the resulting saved states and performed one optimization step, i.e calculated the maximum likelihood estimates of all parameters when adding a state change on a given edge to the saved state. As shown in figure 1, we measured both the speed-up resulting from using the faster option and the difference in the maximum log likelihood found.

As expected the speed-up achieved increases with the number of states already present in the tested configuration. At 5 state changes, the fast optimization is on average 10 times faster than the regular one, with a number of outliers with speed-ups of up to 50 times. At 15 state changes the speed-up is of 70 times on average, a considerable improvement. The difference in the maximum log-likelihood obtained using the less-precise fast option also increases with the number of state changes, although the difference remains small compared to the log-likelihood value, which is on average -1690 for the regular optimization across all categories. The runtimes for one edge are on average 170s at 5 state changes and 1250s at 15 state changes for the regular optimization. Since every step of the algorithm involves testing all edges of the tree, the "fast" option is thus necessary to ensure completion of the inference on trees with more than 10 clusters.

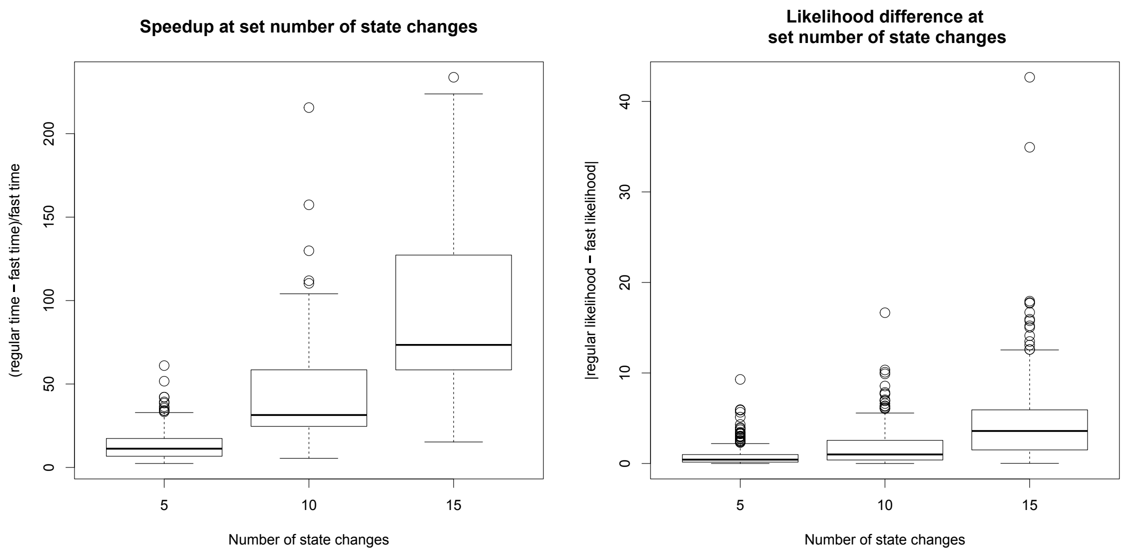

Figure 1: Box plots representing the speed-up (A) and likelihood difference (B) on one step of the algorithm when using the "fast optimization" option compared to the default settings. The dataset used was divided in three categories based on the number of state changes already found by the inference before the test was run.

6 Parameter inference with incomplete sampling

Tables 4 and 5 show the results of the MSBD inference on simulated trees with respectively $\sigma = 0.75$ and $\sigma = 0.5$. The sampling proportions were fixed to the correct values in the inference. As we can see, lower sampling proportions lead to lower accuracy in the parameter estimates, in particular in the transmission rate estimates. However, the relative error remains low, and the MSBD estimates appear to remain reliable even with lower sampling proportions, as long as the actual sampling proportion is known.

Dataset parameters		$\lambda_0 = 35, z = 12, \mu = 1, \gamma = 0$	$\lambda_0 = 35, z = 15, \mu = 1, \gamma = 0$	$\lambda_0 = 15, z = 1, \mu = 5, \gamma = 0.5$	$\lambda_0 = 15, z = 2, \mu = 5, \gamma = 0.5$
Average number of clusters	simulated	1	1	4.50	5.56
	$>$ 5 individuals, simulated	1	1	1.70	2.12
	inferred	1.55	1.41	3.86	3.85
Average transmission rate	simulated	1.90	1.35	11.20	8.65
	inferred	2.38	1.83	11.43	8.92
	median absolute error	0.36	0.42	0.82	0.73
Average removal rate	simulated	1.0	1.0	5.0	5.0
	inferred	0.89	0.94	5.07	4.82
	median absolute error	0.25	0.23	0.86	0.81

Table 4: Parameter inference on several datasets with extinct sampling $\sigma = 0.75$. Each dataset contains 200 trees of 50 tips each, simulated under a multi-state birth-death process using Gillespie's algorithm. Transmission rates are averaged over the entire tree.

Dataset parameters		$\lambda_0 = 50, z = 12, \mu = 1, \gamma = 0$	$\lambda_0 = 50, z = 15, \mu = 1, \gamma = 0$	$\lambda_0 = 20, z = 1, \mu = 5, \gamma = 0.5$	$\lambda_0 = 20, z = 2, \mu = 5, \gamma = 0.5$
Average number of clusters	simulated	1	1	4.39	5.29
	$>$ 5 individuals, simulated	1	1	1.37	1.84
	inferred	1.48	1.37	3.03	3.21
Average transmission rate	simulated	3.67	2.39	15.94	12.66
	inferred	4.09	2.91	16.62	13.13
	median absolute error	0.30	0.39	1.18	1.03
Average removal rate	simulated	1.0	1.0	5.0	5.0
	inferred	0.93	0.89	4.96	4.86
	median absolute error	0.28	0.26	1.07	0.96

Table 5: Parameter inference on several datasets with extinct sampling $\sigma = 0.5$. Each dataset contains 200 trees of 50 tips each, simulated under a multi-state birth-death process using Gillespie's algorithm. Transmission rates are averaged over the entire tree.

7 Supplementary figures

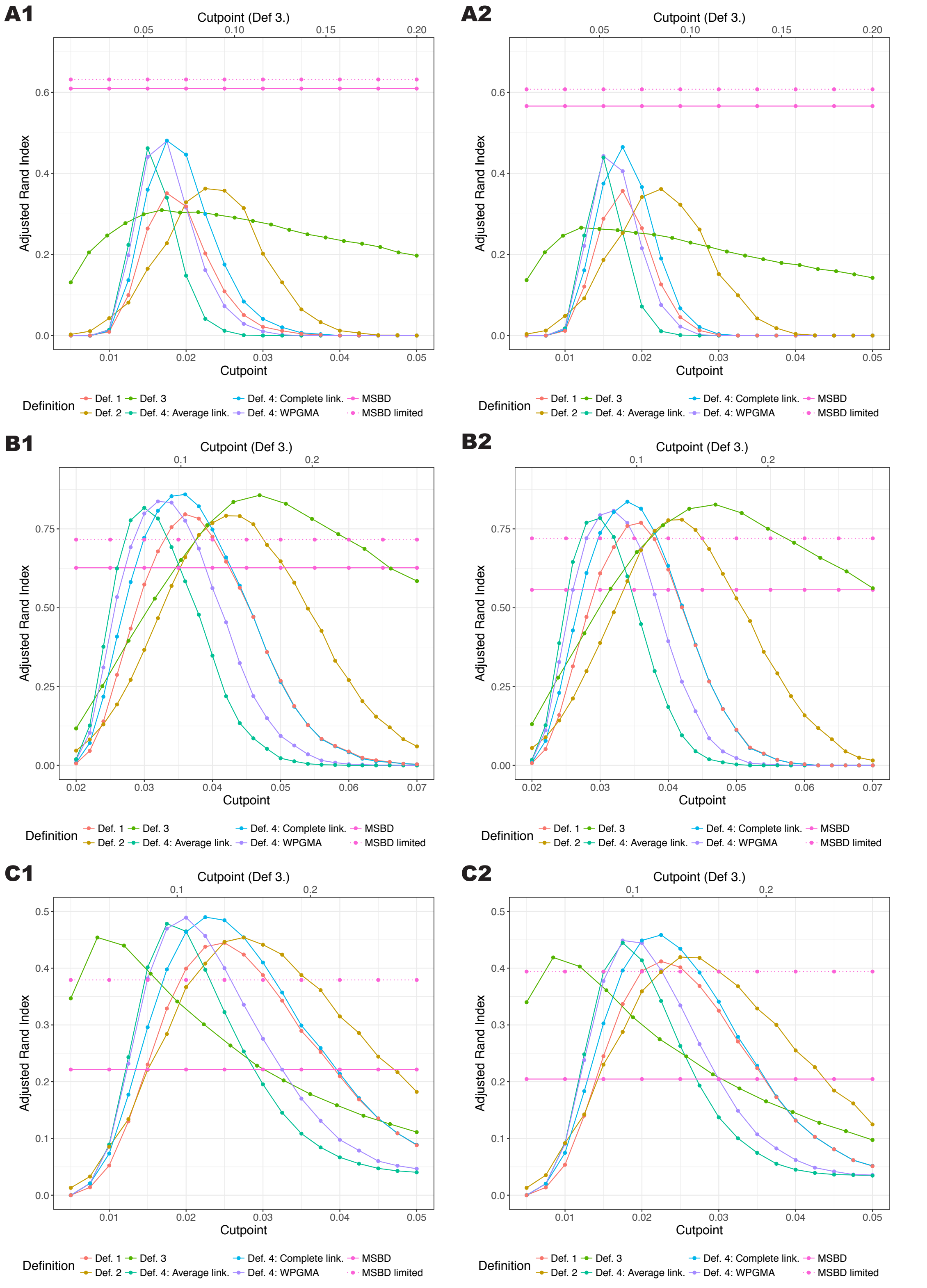

Figure 2: Comparison of the average ARI obtained by the different clustering methods in function of the set cutpoint on networks A (parts A1,A2), B (parts B1,B2) and C (parts C1,C2). For each network the first column (part 1) shows the results for weight $w = 0.5$ and the second column (part 2) for $w = 0.75$. Our proposed MSBD method is not dependent on a cutpoint. The cutpoint range for Definition 3. is shown on the x-axis on the top, the cutpoint range for all other definitions are shown on the x-axis at the bottom.

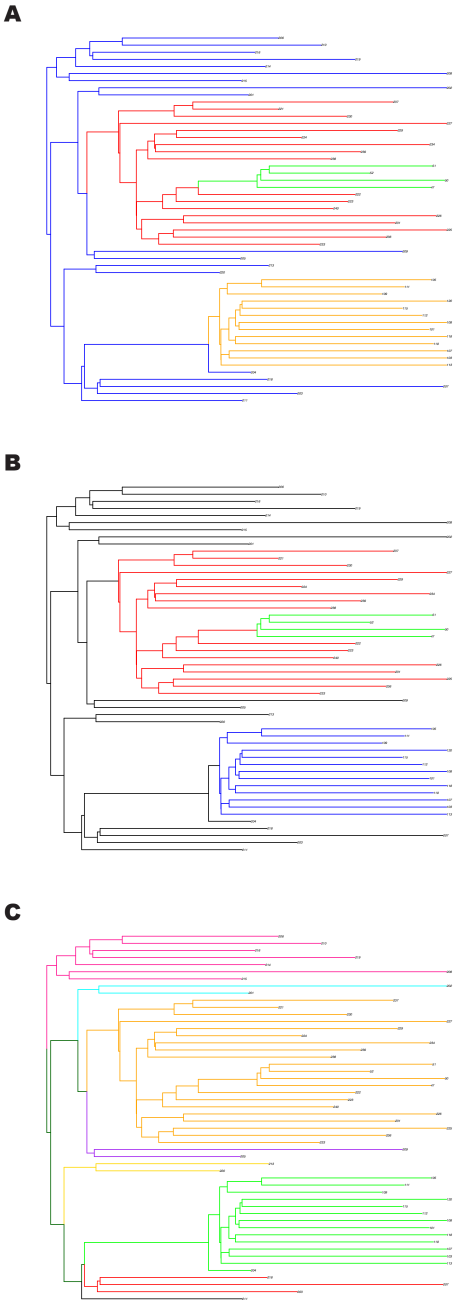

Figure 3: Illustration of nested cluster inference on a tree produced by a type A network. Part A shows the true clustering, part B shows the clustering inferred by MSBD and part C shows the clustering inferred by the cutpoint method following Definition 1 with a cutpoint of 0.02.

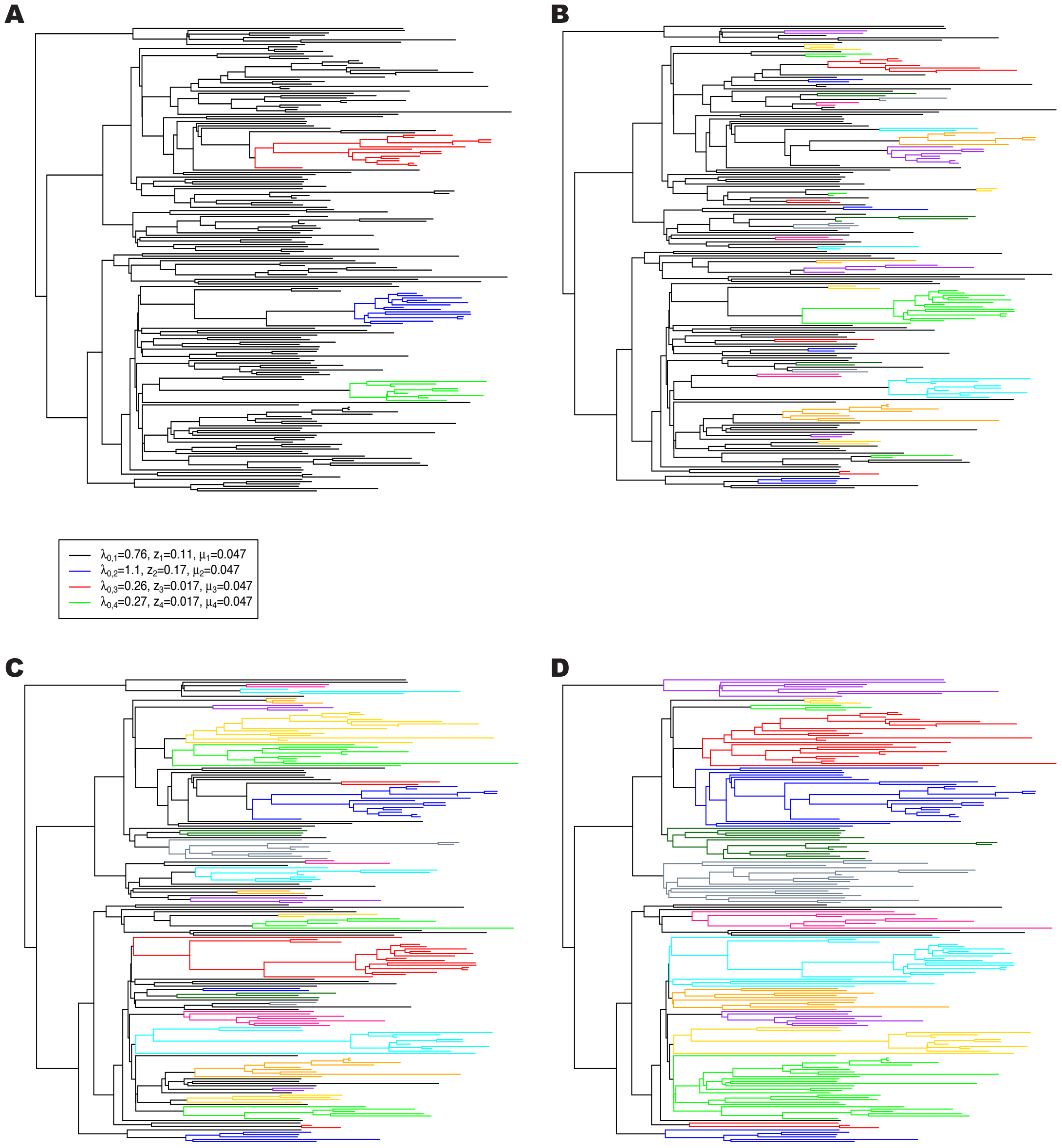

Figure 4: Analysis of the empirical HIV tree. Comparison of the clusters obtained with MSBD (part A) or with PhyloPart with a bootstrap threshold of 0.0 and a genetic distance threshold of 0.01 (part B), 0.02 (part C) and 0.1 (part D).

References

[1] Rasmussen DA, Kouyos R, Günthard HF, Stadler T. Phylodynamics on local sexual contact networks. PLOS Computational Biology. 2017 03;13(3):1–23.
